# Auricular composite tissue graft for the correction of alar rim defect in Asian patients: a retrospective case series of 15 patients

**DOI:** 10.3389/fmed.2025.1710063

**Published:** 2026-01-21

**Authors:** Xinyi Feng, Baofu Yu, Qifeng Wu, Jinguang He, Qimin Zhou, Yangxuanyu Yan, Jiao Wei, Chuanchang Dai

**Affiliations:** 1 Department of Plastic and Reconstructive Surgery, Shanghai Ninth People’s Hospital, Shanghai Jiao Tong University School of Medicine, Shanghai, China; 2 Department of Ear, Nose and Throat, Shanghai Ninth People’s Hospital Huangpu Branch, Shanghai Jiao Tong University School of Medicine, Shanghai, China

**Keywords:** alar rim defect, Asian patients, auricular composite tissue, rhinoplasty, transplantation

## Abstract

**Background:**

Various procedures can now be utilized for the correction of alar rim defect, most notably alar rim grafts and local flap transplantation. However, each of these procedures possesses inherent limitations. In this retrospective case series, we present a novel approach using auricular composite tissue grafting for the treatment of alar rim defect.

**Methods:**

A retrospective analysis was conducted on 15 patients who underwent auricular composite tissue grafting. The surgical procedure included incision, release of nasal alar mucosa surrounding the alar rim, and subsequently harvest and transplantation of composite tissue from the antihelix region to the defect area. Surgical outcomes were assessed by standardized photographic measurements of alar height and a previously validated patient satisfaction questionnaire.

**Results:**

No postoperative local necrosis, infection, or delayed wound healing occurred in any patients. Three patients underwent secondary surgery to achieve an enhanced aesthetic results. Mild contraction and scar color mismatch were observed at 3–6 months after the operation. By 1 year, the scars were almost indistinguishable. During long-term follow-up, two patients presented mild retraction of the graft. Overall, 87% of the patients were satisfied with the outcome of the surgery.

**Conclusion:**

Auricular composite tissue grafting appears to be a feasible technique for the repair of alar rim deformities, providing satisfactory aesthetic outcomes and high patient satisfaction.

## Introduction

Alar rim defect is a common type of alar deformity in which the alar rim is displaced posteriorly and the external nostril is exposed. This condition arises from various etiologies, most commonly congenital causes, trauma, and rhinoplasty complications. Such complications can include disruption of muscular attachment points, excessive skin excision, over-resection of the lateral crus, and infection (1). These circumstances can result in reduced cartilaginous support, muscle denervation, and scar contracture (2). In a few instances, congenital abnormalities in alar cartilage development may also be responsible for the condition (3). Alar rim defect can compromise nasal contour and lead to external nasal valve dysfunction, thereby affecting both appearance and breathing.

Several classification systems are used to describe the alar rim defects. The Gunter classification is based on alar-columellar discrepancies and defines a defect as present when the distance between the alar rim and the long axis of the nostril exceeds 2 mm on the lateral view (4). From the frontal perspective, alar rim defects are commonly categorized as lateral, medial, and central types (5, 6). In Asian populations, an additional classification has been described according to the degree of nostril exposure on the frontal view (7). Because the alar structure consists of cartilage, oriented muscle, and layers of elastic fibers, therapeutic measures must address both static structural support and dynamic respiratory function, which presents an enormous challenge (2).

Several approaches are presently used to treat alar rim defect, including alar rim grafts, flap transplantation, and alar spreader grafts, each with certain shortcomings. Alar rim grafts are primarily utilized for mild to moderate defects or when the soft tissue triangle is compromised, but they often necessitate additional surgical interventions when skin contracture or lining deficiencies are present (8, 9). Flap transplantation, especially rotation of nasal flaps utilizing external Z-plasty, is an effective method for alleviating skin contracture but is most frequently for defects larger than 4 mm, particularly when they are accompanied by skin or mucosal deficiency. However, this technique may result in conspicuous scarring (10–12). Alar spreader grafts are used for patients with external valve collapse or moderate defects due to shortened lateral crus, but they inadvertently add volume to the nasal tip at the expense of aesthetics (6). Other operations, such as lateral crural grafts, have also been reported (13, 14). These techniques are unlikely to be universally applied to all types of alar rim defect, and their limitations are particularly evident in Asian patients with thicker alar soft tissue and a relatively rigid skin envelope. Therefore, research into new and more appropriate means of compensating for alar rim defect is a vital area for nasal plastic surgeons.

This study presents an innovative application of auricular composite tissue grafting for the repair of alar rim defect, emphasizing its functional and aesthetic outcomes. Its advantages include restoration of structural support, improved cartilage stability, and high graft survival. During an extended follow-up period, we evaluated the surgical correction of the alar rim defect and patient aesthetic satisfaction.

## Patients and methods

A retrospective clinical study was conducted to evaluate postoperative characteristics, surgical outcomes, and complications of alar rim reconstruction using autologous auricular composite tissue graft in all patients who presented with alar rim defect from January 2019 to December 2023. This study was conducted based on the Declaration of Helsinki and presented according to STROBE guidelines for observational studies after approval by the local ethics committee. All patients provided written informed consent for both the surgical procedures and the use of anonymized clinical data for research purposes.

All the patients exhibited an extensive alar rim defect, and nasal CT scanning was performed to ensure posterior nasal structure integrity. The degree of defect was graded according to the Kim classification system for frontal analysis and the Gunter classification system for profile analysis. The condition of the skin and cartilage at the auricular region was also assessed to exclude contraindications such as active infection in the donor or recipient sites, serious systemic diseases, or prior history of radiation therapy.

### Surgical techniques

Chondrocutaneous composite grafts from the ear are a common option for nasal defect reconstruction, as they offer sufficient structural rigidity (15). Compared with the conchal bowl (16), the antihelix was selected as the donor site because its natural curvature closely matches the contour of the alar rim, allowing minimal graft reshaping and more favorable pliability. The skin thickness and sebaceous gland density in this region also resemble those of the nasal alar, providing improved color-texture compatibility.

The procedure was conducted under general anesthesia, and the donor site was marked with Meilan reagent. Proper assessment of the alar rim defect was carried out, followed by precise delineation of the defect boundaries. Scar tissue excision was undertaken to alleviate the alar contracture, creating a well-vascularized wound bed for enhanced graft viability.

A composite graft of equal size was designed on the auricle and contoured to match the curvature and dimensions of the alar rim defect. The graft was harvested to include antihelical cartilage and full-thickness skin with the underlying fibrofatty subcutaneous layer, while preserving the auricular perichondrium and its vascular network. The donor site was closed using 5–0 absorbable sutures on a corner needle (Ethicon, USA), with careful approximation of the perichondrial plane to prevent auricular deformity.

**Figure 1:**
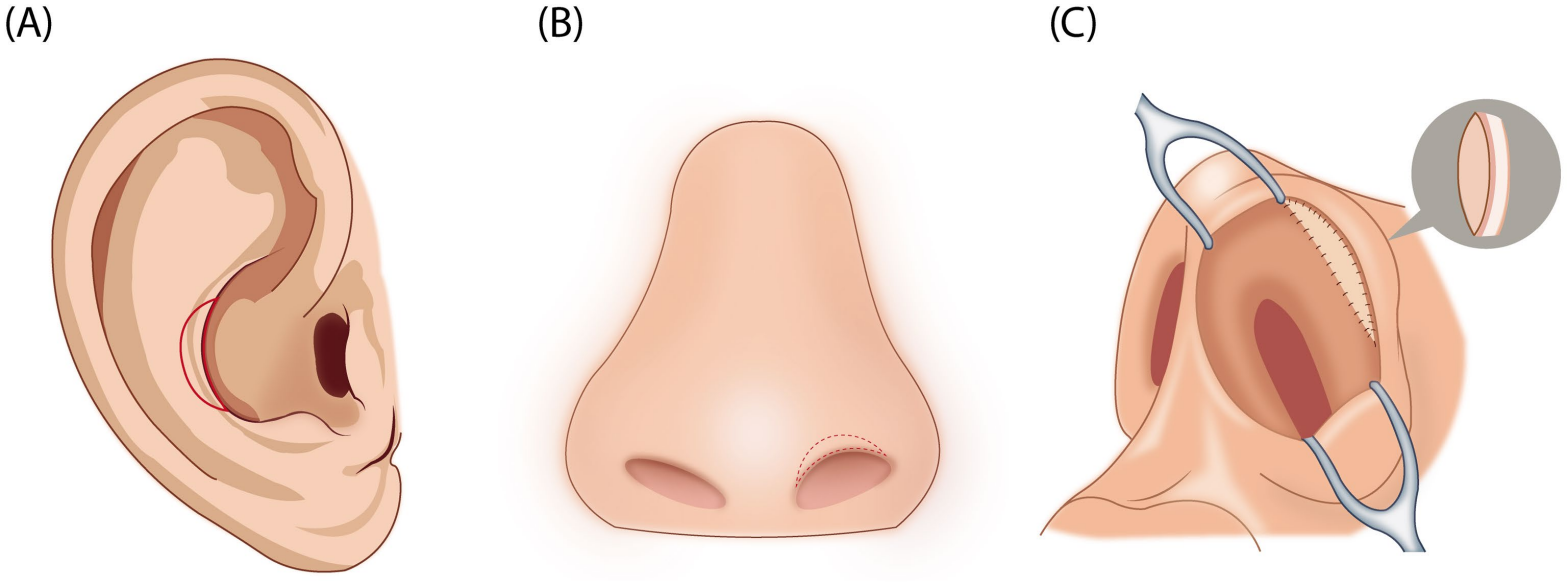
Key surgical steps in auricular composite tissue graft. **(A)** Design and marking of the donor graft on the auricle, tailored to match the contour and size of the alar defect. **(B)** Marking the recipient site with Meilan reagent, followed by incision and release of the contracted skin. **(C)** Transfer and fixation of the auricular composite tissue to the retraction site of the nasal alar; a lightly compressed sponge was applied to enhance graft adherence and integration.

The auricular composite tissue was gently transferred to the defect site and secured with 5-0 absorbable sutures to eliminate dead space and promote graft adherence. Additional epidermal approximation was achieved using 6-0 PDS sutures on a tapered needle (Ethicon, USA) to ensure precise epidermal alignment. A minimally compressed, inflated sponge was placed over the graft to enhance graft integration with the recipient site (Figure 1).

Given the high concentration of sebaceous glands at the graft site, meticulous post-surgical wound care regimens were adopted to promote graft integration and minimize complication risks. Patients were prescribed a 3-day course of postoperative broad-spectrum oral antibiotics in addition to graft site irrigation with saline to lower the risk of infection. Following suture removal on the seventh day, silicone gel therapy was initiated for 6 months to mitigate scar formation. Patients were advised to avoid sun exposure, smoking, alcohol, and spicy foods for at least 6 months.

### Postoperative evaluation

Routine postoperative monitoring was conducted, and any complications were systematically documented and analyzed. For objective assessment of surgical outcomes, standardized photographs of each patient’s face in frontal, profile, and basal views were taken at each visit before and after surgery to assess scar formation and secondary retraction. A series of measurements was systematically recorded, including preoperative alar rim defect height, postoperative correction, comparative measurements between affected and healthy sides, as well as postoperative scar formation and graft retraction during the follow-up period. Moreover, to evaluate postoperative graft retraction, standardized frontal and lateral view photographs taken at 1 month and 12 months postoperatively were compared. Mild or minimal retraction was defined as an upward retraction of the alar rim midpoint greater than 1 mm on the 12-month photograph, which corresponded to a slight increase in nostril exposure on the frontal view.

Subjective surgical outcomes were evaluated by quantifying patient satisfaction during each follow-up appointment using a previously reported questionnaire (17). Responses were categorized into four groups: very satisfactory, mostly satisfactory, unsatisfactory, and very unsatisfactory, depending on functional improvement and aesthetic outcomes (17).

## Results

This study enrolled 15 patients (4 males, 11 females), all of whom were East Asian, with a mean age of 25 ± 2.7 years (range, 20–33 years). Etiologies of alar rim defect included congenital deformity (*n* = 6), trauma (*n* = 4), and rhinoplasty-associated complications (*n* = 5). Additional patient characteristics, such as smoking status, diabetes or other systemic disease, prior radiation exposure, and use of anticoagulant or immunosuppressive medications, are summarized in Table 1. All procedures were completed uneventfully, with a mean operative time of 1.4 ± 0.6 h. Patients were followed for 12–25 months, with a mean of 18 months.

**Table 1:** Summary of patient characteristics.

Variable	Value
Total patients	15
Age, mean ± SD (range)	25 ± 2.7 (20–33)
Sex, *n* (%)	Male (*n* = 4, 27%), Female (*n* = 11, 73%)
Etiology, *n* (%)	Congenital deformity (*n* = 6, 40%) Trauma (*n* = 4, 27%) Rhinoplasty-associated complications (*n* = 5, 33%)
Smoking, *n* (%)	3 (20%)
Diabetes, *n* (%)	2 (13.3%)
Other systemic disease, *n* (%)	0 (0%)
Prior radiation history, *n* (%)	0 (0%)
Anticoagulant use, *n* (%)	0 (0%)
Immunosuppressive medications, *n* (%)	0 (0%)

Standardized photographic measurements were available in frontal, profile, and basal views for all 15 patients. Using the alar rim midpoint as the reference point, the mean preoperative alar height was 4.2 ± 0.9 mm, which decreased to 2.1 ± 0.6 mm postoperatively, with an average improvement of 2.1 mm on the frontal view. Mild, measurable retraction was observed in two patients, and the degree of retraction remained stable beyond 12 months postoperatively. No cases of moderate or severe retraction occurred.

Postoperative assessment revealed that there was no necrosis of the auricular composite graft nor any cases of infection or delayed healing among the patients. The auricle incision healed well, with only a superficial notch on the auricle in some patients. The alar wound healed rapidly by primary intention within 2 weeks, and recipient-site scars developed mild cicatricial contraction and color discrepancies over the 3–6 months postoperative period. Notably, the structural integrity of the transplanted cartilage framework was maintained, with no evidence of warping or resorption observed in serial photographic assessments. Most scars became virtually imperceptible within 1 year of surgery. Long-term follow-up demonstrated that some grafts experienced minimal retraction that did not compromise overall appearance. Three patients received secondary auricular composite grafting to further refine aesthetic outcomes. 87% of the patients rated their outcome as satisfactory (Table 2).

**Table 2:** Patient satisfaction evaluation postoperatively based on a satisfaction survey.

Response	No. patients	Percentage (%)	Satisfaction score (mean ± SD)
Very satisfactory	9	60%	9.1 ± 0.7
Mostly satisfactory	4	27%	7.3 ± 0.5
Unsatisfactory	2	13%	4.0 ± 0.0
Very unsatisfactory	0	0%	–
Total patients	15	100%	7.8 ± 1.9

Satisfaction scores were defined as follows: Very satisfactory = 9–10; Mostly satisfactory = 7–8; Unsatisfactory = 4–6; Very unsatisfactory = 0–3.

### Typical case reports

#### Case 1

**Figure 2:**
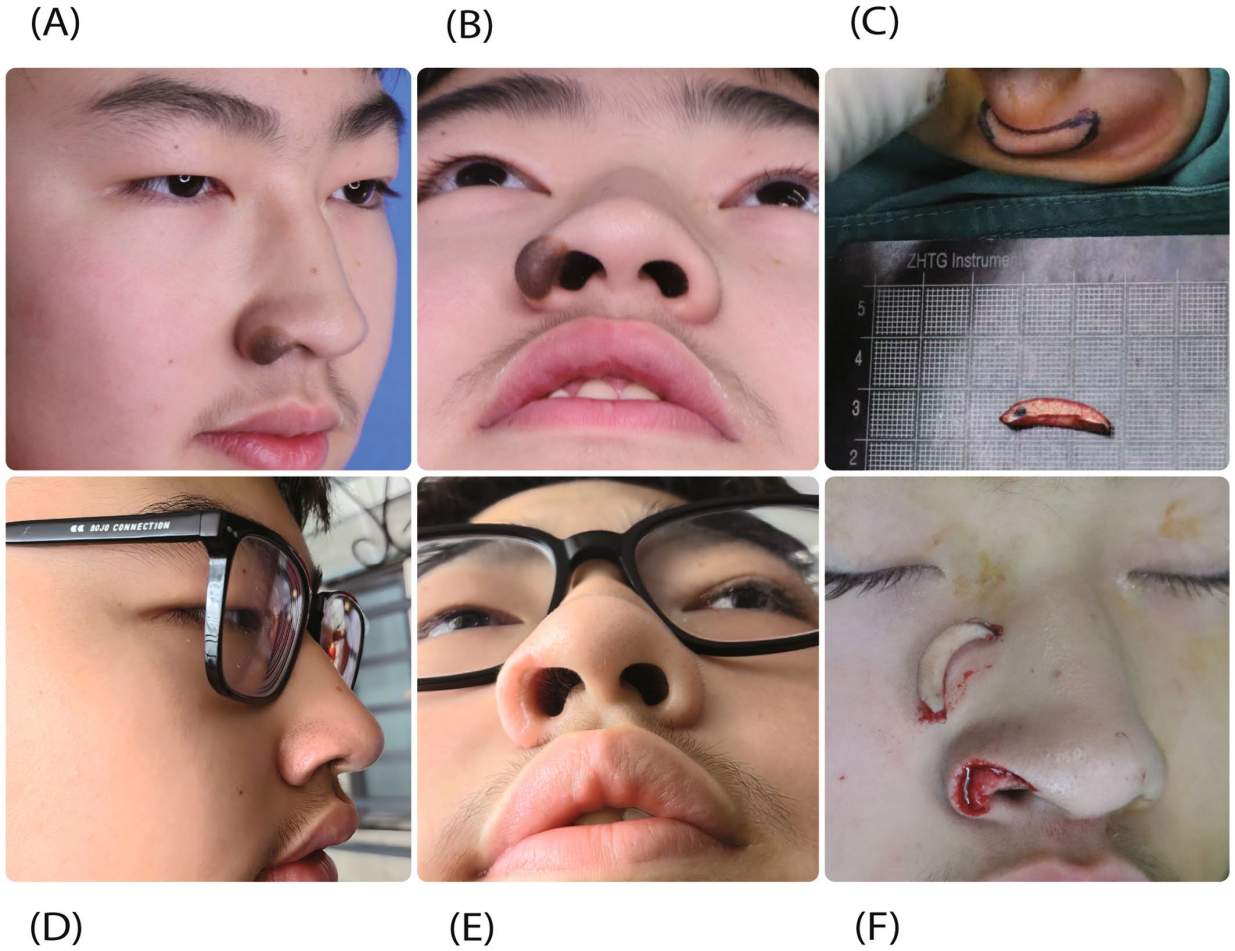
A 17-year-old male patient underwent auricular composite tissue graft for correction of iatrogenic alar rim defect following nevus excision. **(A,B)** Preoperative assessments reveal a nevus on the right alar rim. **(C,F)** Intraoperative photographs illustrate the right alar rim defect and demonstrate the excellent adaptation of the composite graft to the recipient bed. **(D,E)** Postoperative results at 3 months showing restored alar contour and graft viability.

A 17-year-old male patient presented for surgical excision of a nevus on the right alar rim and reconstruction of the resulting defect. The procedure was performed using auricular composite tissue graft. At 3 months of postoperative observation, the graft was well-integrated with excellent improvement in the contour and support of the alar rim. The graft was viable and stable without any evidence of necrosis or excessive contraction (Figure 2).

#### Case 2

**Figure 3:**
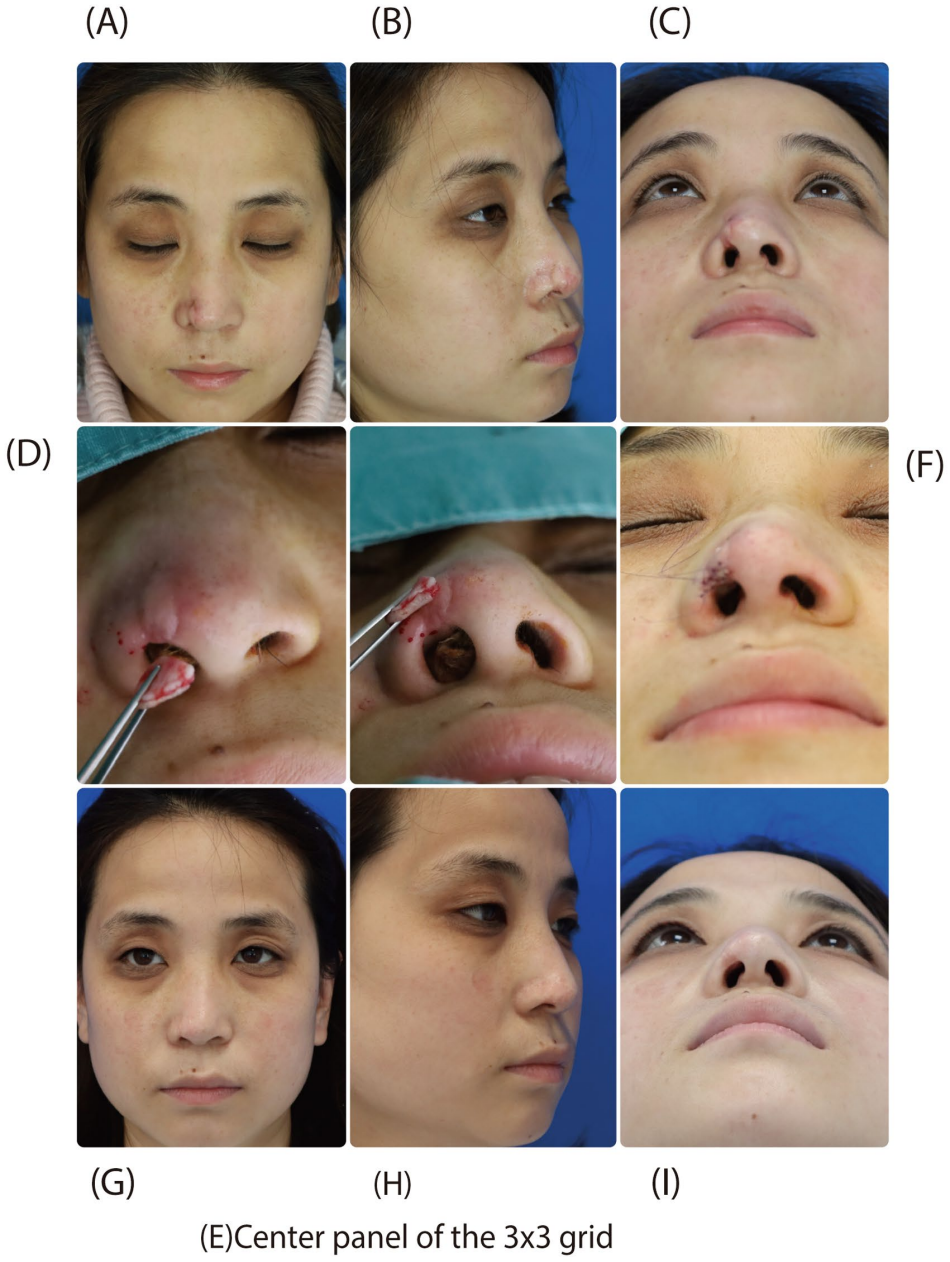
A 41-year-old female patient received auricular composite tissue graft for correction of unilateral alar defect. **(A–C)** Preoperative views show scar formation and slight defect at the right alar rim. **(D–F)** Intraoperative schematic diagrams illustrate the contouring and thickness matching of the auricular composite tissue to the defect. **(G–I)** Three-month follow-up reveals excellent graft integration with no visible scarring and optimal aesthetic restoration of the alar rim.

A 41-year-old female patient presented with a right alar rim defect due to scar contracture following post-rhinoplasty infection. The patient sought surgical treatment for aesthetic restoration of the nasal contour. The procedure involved thorough release and excision of the scar tissue, soft tissue defect reconstruction using a fascia-based island flap, and correction of the alar rim defect using auricular composite tissue graft. Staged operations resulted in a significant improvement in nasal appearance. The graft had incorporated well with surrounding tissues and maintained stable morphology on 3-month follow-up. All transplanted tissues remained well-vascularized without complications such as infection, necrosis, or disturbed wound healing (Figure 3).

## Discussion

Alar rim defect causes nasal aesthetical disruption by exposing excessive nostril surface area, which induces airflow turbulence and frequently leads to patient anxiety (18). With the increasing prevalence of rhinoplasty, iatrogenic alar rim defect is becoming increasingly common. The primary etiological factors are structural insufficiency of the lateral crus cartilage, including congenital cartilage dysplasia, cartilage injury from trauma, and excessive resection of the lateral crus in rhinoplasty (1, 3). Notably, East Asians exhibit a higher vulnerability to surgical or traumatic complications due to the inherent fragility of their nasal cartilage, which predisposes them to alar rim defect (19). Given the anatomical complexity of the alar region, which consists of a hierarchical arrangement of elastic fibers, cartilage, and skin, repair ideally needs to replicate this hierarchical structure (2). In addition, correction of both dynamic and static changes with maintenance of aesthetic uniformity is crucial, especially in color harmony with skin and natural touch perception. In order to prevent secondary retraction, adequate architectural support is needed at the graft site, presenting considerable difficulty for the rhinoplasty surgeon.

Auricular chondrocutaneous composite grafts are widely used for reconstructing nasal defects, including those exceeding 1.5 cm (15). The conchal bowl is a conventional donor site (16). Previous studies have also demonstrated the efficacy of superior antihelix composite tissue in reconstructing large, thick alar defects and reestablishing the alar nostril ring with successful functional and cosmetic results (20, 21). The advantages of using this tissue are evident, given its similarity to nasal tissue in terms of thickness, color, and sebaceous gland density, as well as higher graft survival rates. The antihelix provides several technical advantages over the conchal bowl or other auricular regions for alar rim reconstruction. In comparison with conchal bowl cartilage or other auricular cartilage, which typically require more extensive trimming, the natural curvature of the antihelix region aligns seamlessly with the arched structure of the alar rim, allowing for easier sculpting during surgery. Antihelix tissue offers superior pliability while providing adequate structural rigidity, and its natural configuration helps prevent distortion (21, 22). The donor site typically showed only a superficial notch, with no donor-site morbidity. For that reason, we aimed to employ this tissue for the treatment of mild alar rim defect.

In this study, we utilized auricular composite tissue to address alar rim defect. Postoperatively, no cases of local necrosis, infection, or delayed healing were encountered, though a minority of patients exhibited mild graft retraction during long-term follow-up. Scarring at the graft site became inconspicuous within 1 year post-surgery. A minority of patients sought secondary surgical correction to achieve better aesthetic outcomes. The efficacy of the auricular composite tissue was evaluated based on preoperative and postoperative nasal measurements, along with patient satisfaction questionnaires. In the study patient population, satisfactory outcomes were observed in 87% of patients with no serious subsequent complications. Follow-up beyond 12 months revealed no new graft failures, substantial late retraction, or contour deterioration, indicating that graft stability is generally achieved within the first postoperative year. These outcomes suggest that the graft is a viable option for alar rim defect management. Validated tools such as ROE or NAFE were not used due to the retrospective design and the lack of standardized preoperative questionnaires. Instead, we used a satisfaction questionnaire published by Zhang et al. (17), supplemented with objective photographic measurements of alar height and retraction to evaluate surgical outcomes.

Compared with conventional techniques, this approach offers considerable advantages. Transplantation of auricular composite tissue allows for the simultaneous transfer of skin, subcutaneous fibrofatty tissue, and cartilage, thus achieving optimal structural compatibility with the recipient’s nose anatomy. The grafts are highly vascular and encourage better integration between recipient and donor sites, enhancing graft survival rates. Moreover, the subcutaneous fibrofatty layer and cartilage contribute to the contour of the alar rim while providing adequate support to prevent graft retraction. The composite graft of auricular cartilage with skin optimally alleviates skin tension and imposes fewer demands on the recipient site’s skin quality and thus allows wider application (23, 24). For patients with mild nasal alar defect, who may be reluctant to accept extensive scarring, the auricular graft provides a solution for a more natural appearance.

During surgery, accurate inset and fixation of the auricular composite graft into the nasal defect are paramount, necessitating meticulous shaping of the graft. A 1:1 ratio of skin to cartilage was maintained, with preservation of the intact interface between the skin and perichondrium to optimize graft viability (25). Graft survival also depends on appropriate graft dimensions that facilitate revascularization. In our practice, the width of the auricular composite graft is typically maintained between 2 and 4 mm, while graft length has no strict limitation and is determined by the curvature and extent of the alar rim defect. This width range is based on institutional experience rather than a strictly evidence-based upper limit.

Sharp dissection techniques were employed to avoid injury to the perichondrium, and electrocautery use was minimized to preserve the vascular network of the composite tissue. Postoperatively, light pressure dressings were applied to maintain consistent contact and promote revascularization of the graft. Given the high density of sebaceous glands at the recipient site, extended antimicrobial therapy was administered to reduce the risk of inflammation and infection.

Some patients developed mild retraction, likely due to inadequate structural support from insufficient cartilage, and they opted for secondary procedures. In these secondary procedures, the graft width was generally maintained at approximately 2 mm, with particular attention to ensuring adequate cartilage width, even slightly exceeding the width of the skin component. The secondary procedures were performed using the same technique as the initial operation. Donor-site morbidity of the ear was minimal; no patients developed contour deformities, and no aesthetic complaints were reported during follow-up.

This study has several limitations. Initially, the sample size was small, and all cases were derived from a single center with relatively homogeneous age and ethnicity. These factors limit the generalizability of the findings to other populations and practice settings. Additionally, no formal assessment of external nasal valve function, including clinical examination, the Cottle maneuver, or patient-reported breathing changes, was performed. These limitations should be considered when interpreting the findings.

In summary, auricular composite tissue graft provides a feasible treatment approach for alar rim defect, as it exhibits structural features similar to nasal tissue, including sebaceous gland distribution and skin color-texture compatibility (22). The grafts demonstrated favorable pliability and a high survival rate. In light of the limitations noted above, future studies should include larger and more diverse patient cohorts and incorporate more objective assessments of external nasal valve function to further improve surgical outcomes.

## Conclusion

In this study, we confirmed that auricular composite tissue is a feasible treatment option for alar rim defect. Quantitative assessments revealed durable improvements in the aesthetic appearance. The transplanted tissue manifested optimal properties, including adequate vascularization, controlled retraction, and structural stability. Our findings provide valuable insights for composite tissue transplantation in other anatomically demanding regions.

## Data Availability

The original contributions presented in the study are included in the article/supplementary material, further inquiries can be directed to the corresponding author/s.
